# Photon-triggered pyroptosis and ferroptosis dual-functional nanoplatform for cancer immunotherapy

**DOI:** 10.1038/s41377-025-01757-6

**Published:** 2025-02-27

**Authors:** Quansheng Cheng, Qingcheng Wang, Songnan Qu

**Affiliations:** 1 Joint Key Laboratory of the Ministry of Education, Institute of Applied Physics and Materials Engineering, University of Macau, Taipa, Macau SAR 999078 China; 2 Department of Physics and Chemistry, Faculty of Science and Technology, University of Macau, Taipa, Macau SAR 999078 China

**Keywords:** Applied optics, Optical techniques

## Abstract

A dual-functional nanoplatform is demonstrated that is found to have the characteristics of cancer cell targeting, pH response, near-infrared fluorescence imaging, and lysosome targeting. It can simultaneously achieve pyroptosis and ferroptosis under the mediation of photons for cancer immunotherapy.

## Abstract (web summary)

A novel strategy for pyroptosis and ferroptosis dual-functional inducers and combined immune activators enhanced photoimmunotherapy.

## Graphical abstract

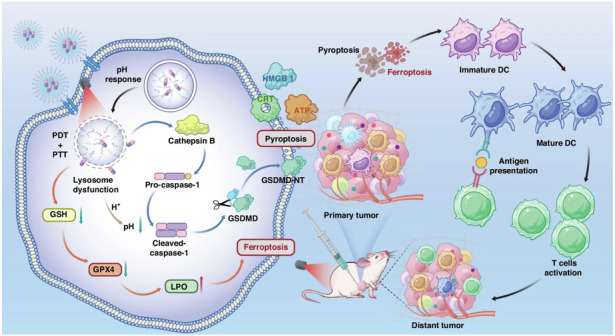

The rapid development of phototherapy, such as photodynamic therapy (PDT), photothermal therapy (PTT) and photocatalytic therapy (PCT), has had a profound impact on the biomedical landscape while laying solid foundation for research and therapeutic development^1–3^. Among the various light-based therapeutic techniques mentioned above, PDT, which utilizes photosensitizer (PS) in the presence of light with a specific wavelength to generate reactive oxygen species (ROS) to irreversibly destroy the targeted tumor tissue, is considered as a promising treatment strategy with various benefits including spatial accuracy, non-invasiveness, and minimal side effects, etc^4^. However, depleting glutathione (GSH) in tumor microenvironment (TME) is still a challenge because the overexpressed concentration level of GSH could prevent generating toxic levels of ROS. In addition, the low immunogenicity of cancer cells and the immunosuppressive TME also limit the immune response induced by PDT. Due to the presence of apoptotic resistance in cancer cells, the therapeutic effect is often limited and unsatisfactory. Therefore, in recent years, the type of cell death induced by PDT promoted by the photothermal effect of nanomaterials has attracted widespread attention^5^.

As immunogenic cell death (ICD) modes, pyroptosis and ferroptosis have demonstrated their potential to induce an acute inflammatory response to produce antitumor immune activity and boost cancer immunotherapy^6^. Generally, ferroptosis is characterized by the depletion of GSH, a reduction in glutathione peroxidase 4 (GPX4) activity, and the failure to metabolize lipid peroxides (LP)^7^. The biology of pyroptosis is characterized by the pore formation in membranes, cellular swelling and lysis, and the release of pro-inflammatory cytokines. More detailed, the cleavage of the gasdermin (GSDM) family can be induced by the activated caspase family to release the GSDM-N structural domain and execute pyroptosis through the pore-forming activity of this structural domain. A large number of inflammatory factors (e.g., IL-1β and IL-18) are released after pore formation, thereby altering the tumor microenvironment, and triggering a strong anti-tumor immune response^8^. As illustrated in Fig. 1, photon-mediated PTT and PDT combined with pyroptosis and ferroptosis are expected to regulate TME and subsequently improve the therapeutic outcomes. At present, the types of PSs that can induce ferroptosis or pyroptosis individually have been widely explored, such as Fe^3+^ phenolics, Bi-Au nanoclusters, GOx-Mn, LaFeO_3_, CaNMs, and metal-organic frameworks^9^. However, in these studies, the introduction of metal species may trigger undesirable toxicity and potentially detrimental effects. Moreover, traditional PSs with planar rigid structures often undergo aggregation-caused quenching (ACQ) when in an aggregated state, thereby promoting non-radiative transitions and reducing ROS yield^10^. Ultimately, ROS-induced ferroptosis or pyroptosis has limited or even no effect. Therefore, designing an efficiently photoinduced pyroptosis and ferroptosis dual-functional theranostic nanoplatform is considered a breakthrough strategy in cancer theranostics.

**Fig. 1:**
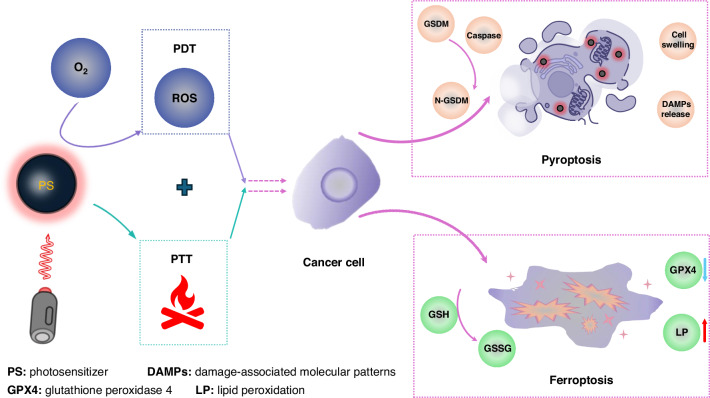
Simple schematic diagram of photon-mediated PTT and PDT combined to induce pyroptosis and ferroptosis

In a newly published paper in Light: Science & Applications, a research team led by Professor Quan Li from Institute of Advanced Materials and School of Chemistry and Chemical Engineering, Southeast University, China and Materials Science Graduate Program, Kent State University, USA, has developed a photon-triggered pyroptosis and ferroptosis dual-functional nanoplatform for cancer immunotherapy^11^. The nanoplatform (M@P) is designed and constructed by self-assembly of aggregation-induced emission photosensitizer MTCN-3 and immunoadjuvant Poly(I: C), which are further encapsulated in amphiphilic polymers. As they have discovered, this nanoplatform is found to have the characteristics of cancer cell targeting, pH response, near-infrared fluorescence imaging, and lysosome targeting. Thus, the M@P nanoplatform targeting lysosomes can generate a large amount of ROS and heat under light irradiation, leading to lysosomal dysfunction and subsequently triggering tumor pyroptosis and ferroptosis (Fig. 2).

**Fig. 2:**
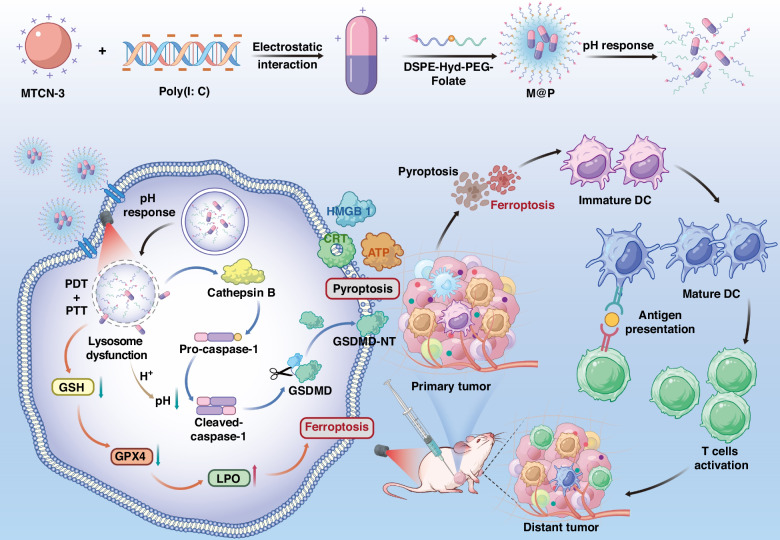
Illustration of multifunctional nanoplatforms M@P inducing cancer cells pyroptosis and ferroptosis for cancer photoimmunotherapy. Note that the figure and caption are reproduced from the original manuscript (Wang et al.^11^)

The above findings can lead to an important reference to cancer photoimmunotherapy in the field of nanomedicine. In fact, in the process of photon-mediated PDT and PTT, the subcellular localization and enrichment of PS or nanoplatforms after being taken up by tumor cells have always attracted much attention. Especially in terms of inducing pyroptosis, most of the reports in the literature focus on mitochondrial-related dysfunction^12,13^. The nanoplatform designed in this article is located in the lysosome, and its pH-responsive characteristics further lead to lysosomal dysfunction, which provides a new perspective for understanding the mechanism of pyroptosis. Moreover, compared with treatment with a single PS, the fluorescence intensity of M@P significantly increases due to the restriction of intramolecular movement of MTCN-3 by poly (I: C). Note that, the combination of MTCN-3 and polymer (I: C) can enhance the efficacy of tumor immunotherapy^14,15^. This also triggers our thinking about the role of rational use of immune adjuvants in promoting pyroptosis.

Stay at the present, photon-triggered dual-functional inducers of pyroptosis and ferroptosis are rare and limited to covalent organic framework^16^ and the nanoplatform described in this paper. Although the two differ in material type, excitation wavelength and molecular mechanism, they both achieve triple-negative 4T1 tumor immunotherapy with enhanced pyroptosis and ferroptosis (Tab. 1). This work provides a novel strategy for achieving pyroptosis-ferroptosis combined with enhanced photoimmunotherapy. Looking forward, research on developing dual-functional inducers of pyroptosis and ferroptosis will be an emerging hotspot. For example, non-metallic carbon dots (CDs), which are ultra-small sized (<10 nm) carbon nanoparticles with excellent biocompatibility, low cytotoxicity, tunable bandgap, and versatile photophysical properties, endowing them distinct advantages in bioimaging and biomedical applications^17–19^. It is foreseeable that the proposed strategy can give reference to other materials (not limited to CDs) for phototherapy applications. This serves to underscore the utility of the nanoplatform design and suggests exciting directions for future pyroptosis-ferroptosis research (Table 1).

**Table 1:** Summary of Photon-triggered pyroptosis and ferroptosis dual-functional inducers for cancer immunotherapy

Material		λ_ex_ (nm)	Subcellular localization	Molecular mechanism	Animal models	Ref.
Category	pH response					
AIEgens-COF	N/A	660/808 nm	N/A	Pyroptosis: PDT/PTT/ROS/Cas3/N-GSDME Ferroptosis: PDT/PTT/ROS/GPX4	4T1 tumor-bearing mouse model/4T1 tumor-bearing dual-flank model	Ref.^16^
(M@P)	Y	520 nm	Lysosome	Pyroptosis: PDT/PTT/ROS/Cas1/N-GSDMD Ferroptosis: PDT/PTT/ROS/GPX4	Unilateral 4T1-tumor-bearing mice/bilateral 4T1-tumor bearing mice model	This work

*Y* yes, *N.A* not available, *Ref.* reference
